# Comprehensive analysis of clinical indications and viral strain variants among patients infected with SARS-CoV-2 in Inner Mongolia, China

**DOI:** 10.1007/s11262-023-01986-0

**Published:** 2023-03-11

**Authors:** Bo Wang, Xiaocong Li, Weili Xiao, Jiangying Zhang, Haitao Ding

**Affiliations:** 1 Department of Laboratory Medicine, Inner Mongolia People’s Hospital, Hohhot, 010000 China; 2 Department of ICU, Inner Mongolia People’s Hospital, Hohhot, 010000 China

**Keywords:** SARS-CoV-2, Whole-genome sequencing, Spike glycoprotein, Mutations

## Abstract

Since the first appearance of severe acute respiratory syndrome coronavirus 2 (SARS-CoV-2) in 2019, the virus is still evolving and mutating until now. In this study, we collected 6 throat swabs from patients who diagnosed with COVID-19 in Inner Mongolia, China, to understand the entry of multiple SARS-CoV-2 variants into Inner Mongolia and analyze the relationships between variants and clinical features observed in infected patients. In addition, we performed a combined analysis of clinical parameters associated with SARS-CoV-2 variants of interest, pedigree analysis, and detection of single-nucleotide polymorphisms. Our results showed that the clinical symptoms were generally mild although some patients demonstrated some degree of liver function abnormalities, and the SARS-CoV-2 strain was related to the Delta variant (B.1.617.2), AY.122 lineage. The epidemiological investigations and clinical manifestations confirmed that the variant exhibits strong transmission, a high viral load, and moderate clinical symptoms. SARS-CoV-2 has undergone extensive mutations in various hosts and countries. Timely monitoring of virus mutation can help to monitor the spread of infection and characterize the diversity of genomic variants, thus limiting future waves of SARS-CoV-2 infection.

**Supplementary Information:**

The online version contains supplementary material available at 10.1007/s11262-023-01986-0.

## Introduction

**Fig. 1:**
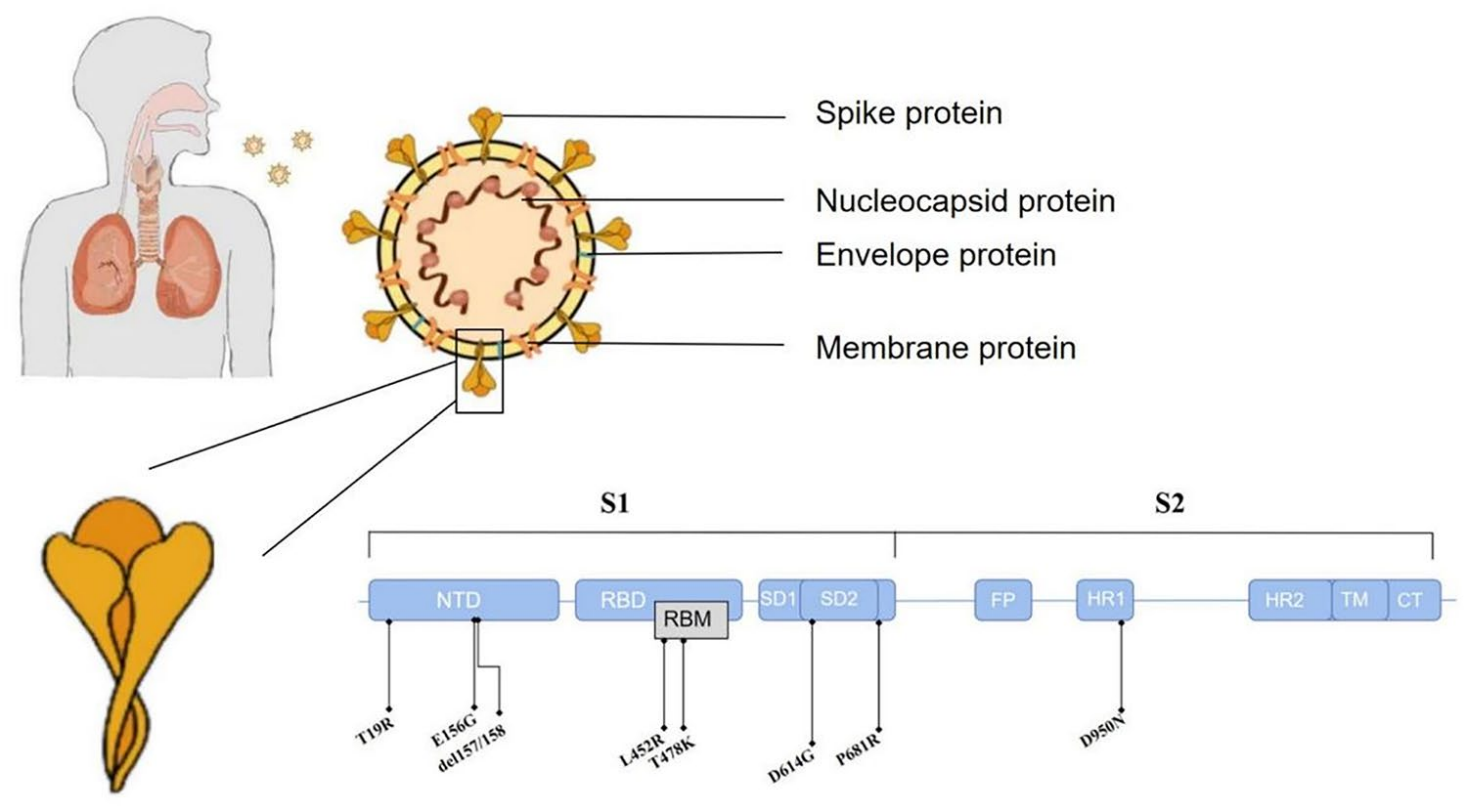
Schematic diagram of SARS-CoV-2 virus structure and main mutation sites of S protein

SARS-CoV-2 began a wave of infections during the Lantern Festival in 2022 in Hohhot, Inner Mongolia. The Hohhot has become the epicenter of viral spread because of the travel and reunion activities for the Spring Festival of (i.e., Chinese New Year) in Inner Mongolia. The emergence of SARS-CoV-2 variants is a key factor in the continued spread of the coronavirus disease 2019 (COVID-19) pandemic. SARS-CoV-2 (Fig. 1) is a pleomorphic, enveloped, positive-sense, single-strand RNA virus [1, 2], its unique Spike protein, receptor binding domain (RBD), bind to the receptor ACE2 on the surface of the target cells and mediates the viral uptake and fusion, which make it have strong infective ability [3, 4]. According to clinical symptoms, COVID-19 can be classified into four types: mild, moderate, severe, and critical. The main clinical symptoms of COVID-19 are fever, fatigue, dry cough, runny nose and nasal congestion, and sometimes patients will appear diarrhea [5, 6]. Patients with basic disease may develop more serious like respiratory distress within 1 week. Severe complications include respiratory stress syndrome, septic shock or metabolic acidosis and coagulation dysfunction [7]. Individual factors such as age and chronic diseases (e.g., hypertension, type 2 diabetes mellitus, and chronic kidney disease) can contribute to disease progression [8, 9].

Thus far, the global SARS-CoV-2 situation remains serious. In the context of the implementation of dynamic zero-COVID and liberalized epidemic prevention policy, the number of infected patients has been another abrupt upsurge, Jilin and Shanghai were a typical example [10]. The current mortality rate is lower than that during the initial outbreak, extensive vaccination efforts worldwide have contributed to the reduction in mortality [11]. By the end of May 2022, 15 SARS-CoV-2 variants had been identified, of which 3 caused of widespread concern; Alpha, Delta, and Omicron (Omicron is considered the main pandemic strain) [12]. Thus, there is a need to monitor genomic variations and evolutionary features of SARS-CoV-2. By the end of March 2022, a total of 436 locally confirmed cases had been admitted to Inner Mongolia hospitals, the age ranged from 1 to 89 years old, the proportion of patients over 60 years old with severe type was higher. During the same period, there were cluster outbreak compared with other provinces in China. In this study, we collected oropharyngeal swabs from six patients who had been diagnosed with COVID-19 in Inner Mongolia, China, to detect the entry of distinct SARS-CoV-2 variants into the locality. We sought to analyze the relationships between variants and clinical features observed in infected patients. Our findings can help to monitor the spread of infection and characterize the diversity of SARS-CoV-2 genomic variants.

## Materials and methods

### Collection of samples

During February and March 2022, six swab samples were collected from patients in the Inner Mongolia People's Hospital who had diagnosed with SARS-CoV-2. Clinical characteristics were collected for all patients. In accordance with the SARS-CoV-2 Diagnosis and Treatment Protocol (Trial Version 8) established by the National Health Commission, PRC, the patients were diagnosed with one of four disease types. Patients with mild disease had mild clinical symptoms and did not exhibit pneumonia during imaging examinations. Patients with common disease had fever and respiratory symptoms, along with visible pneumonia during imaging examinations. Patients with severe disease exhibited any of the following conditions: shortness of breath (i.e., respiratory rate ≥ 30 times/min); oxygen saturation ≤ 93% at rest; arterial partial pressure of oxygen/oxygen concentration ≤ 300 mmHg; and progressive worsening of the clinical symptoms, with lung images that revealed > 50% lesion progression within 24 to 48 h. Patients with critical disease exhibited any of the following conditions: respiratory failure requiring mechanical ventilation, appearance of shock, and concurrent non-respiratory organ failure requiring intensive care unit monitoring and treatment.

### Whole-genome sequencing

Total RNA was extracted from the swab samples using a nucleic acid extraction kit (Bio-Germ, China), and amplified by multiplex polymerase chain reaction (PCR) according to the scheme recommended by WHO [13]. A purification kit (Matridx, China) was used to purify the multiplex amplification products. DNA sequencing libraries were constructed in accordance with the protocol provided with the DNA Library Preparation Kit. The libraries were diluted to enable the calculation of pooled values by real-time quantitative PCR. Each library volume was set to 20 µL (total nucleic acid content of 10–300 ng), and whole-genome deep sequencing was performed using the NGS NextSeq550 AR System (Illumina, America). The raw data was output as FASTQ format, and bioinformatics analysis was used to evaluated the quality of the library. The first virus isolated from Wuhan (Genebank accession number: NC_045512.2, GISAID accession ID: EPI_ISL_402125) was used as the reference strain for the identification of single-nucleotide polymorphisms in our sequence data. Megahit was used to perform the genome assembly, and kma was used to compare the filtered reads with reference genome, and the depth and coverage of the marked reads were counted. Additionally, 1123 high-quality SARS-CoV-2 genomes (S1 Table) from different periods and different countries were downloaded from the public GISAID database, all virus sequence information is obtained from the GISAID (https://gisaid.org). MUMmer method [14] was used to compare the genome sequence of the assembled file, and obtain the mutation information. The matching parameter “mum” was used to obtain the unique maximum matching number of the two sequences, this method is suitable for genome comparison of multiple related species. FastTree was used based on the maximum likelihood method to build the evolution tree to calculate the evolution distance, and the default JC (Jukes-Cantor) model was used. The calculated evolution tree file was uploaded to iTOL (http://itol.embl.de/) tool for graphical display. Further confirmation of subvariant information was performed through a comparison of each sample with two subvariants on the Cov-Lineages website (https://cov-lineages.org/), specific mutations were analyzed to identify viral variant lineages. FASTQ files were analyzed by the ARTIC [15] bioinformatics pipeline to generate consensus sequences.

## Results

### Clinical characteristics of the study participants

**Fig. 2:**
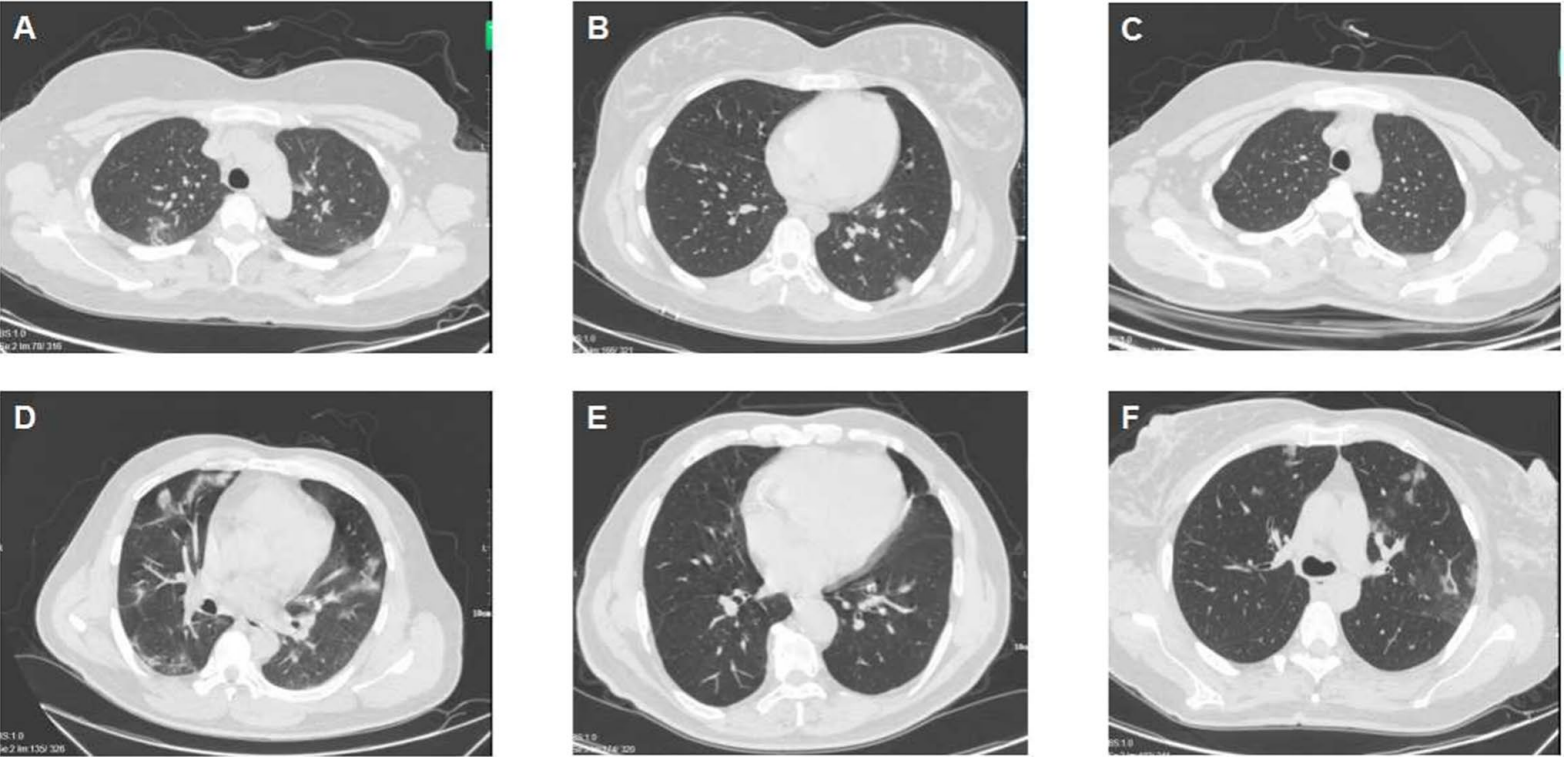
Chest computed tomography scans of six patients with SARS-CoV-2 infection in Inner Mongolia during the Spring Festival of 2022. **A** Case 1: Mild interstitial changes are present in the lower lobe of both lungs, along with multiple ground-glass opacities; **B** Case 2: Multiple ground-glass opacities are present in the lower lobe of both lungs, along with ground-glass nodules in the dorsal segment of the lower lobe of the left lung; **C** Case 3: Solid nodules are visible in the dorsal segment of the lower lobe of the left lung, along with ground-glass nodules in the posterior segment of the upper lobe of the left lung; **D** Case 4: Multiple patchy, nodular ground-glass density shadows are present in both lungs; **E** Case 5: Dense shadows of ground-glass opacity and linear opacities are present under the pleura in the lower lobe of both lungs, along with in the basal segment of the lower lobe of the right lung; **F** Case 6: Multiple patchy consolidation shadows and ground-glass density are present in the posterior apex and lingual segments of the upper lobe of the left lung and the middle lobe of the right lung, calculi are also present in the right kidney

The basic demographic and clinical characteristics of the six patients are shown in Table 1. There were four women and two men, aged 15–69 years. The results of multiple clinical examinations are shown in S2 Table. Although comprehensive data were collected regarding the patients’ immunological indicators, no systematic analysis was performed because of the small sample size and considerable differences among individuals within the group. Patients were classified in accordance with the diagnosis and treatment protocol, while most patients had mild symptoms, one patient was diagnosed with moderately severe disease because of underlying chronic diseases (i.e., hypertension and coronary heart disease). All these patients were cured within 20 days, there were no deaths. The patients performed chest computed tomography on the first day after being diagnosis (Fig. 2), it showed that some ground-glass shadows and mild interstitial changes appear in the lungs of these patients, suggesting that infection with COVID-19 will cause lung injury, which is also a detection method to diagnose COVID-19.

**Table 1:** Basic demographic and clinical characteristics of six patients with SARS-CoV-2 infection in Inner Mongolia during the Spring Festival of 2022

	Case 1	Case 2	Case 3	Case 4	Case 5	Case 6
Sex	Female	Female	Female	Male	Male	Female
Age (years)	34	15	18	44	69	38
Height (cm)	159	175	163	168	169	175
Weight (kg)	69	73	55	76	72	81
BMI (kg/m^2^)	27.3	23.8	20.7	26.9	25.2	26.4
Ct values	18	12	22	19	21	33
Clinical symptoms						
Fever (> 37.3 °C)	Yes	Yes	Yes	Yes	No	No
Cough	Yes	Yes	Yes	Yes	Yes	Yes
Sputum	Yes	Yes	No	Yes	No	No
Sore throat	No	Yes	No	No	No	No
Diarrhea	No	No	No	Yes	No	No
Diminished sense of smell and taste	No	No	Yes	Yes	No	No
Past medical history						
Hypertension	No	No	No	No	Yes	No
Diabetes	No	No	No	Yes	No	No
Coronary heart disease	No	No	No	No	Yes	No
Allergy	Yes	No	No	No	Yes	No
Chronic hepatitis	No	No	No	No	No	No
Length of stay (days)	18	10	9	19	12	12

### Next-generation sequencing and bioinformatics analysis of new SARS-CoV-2 isolates

**Fig. 3:**
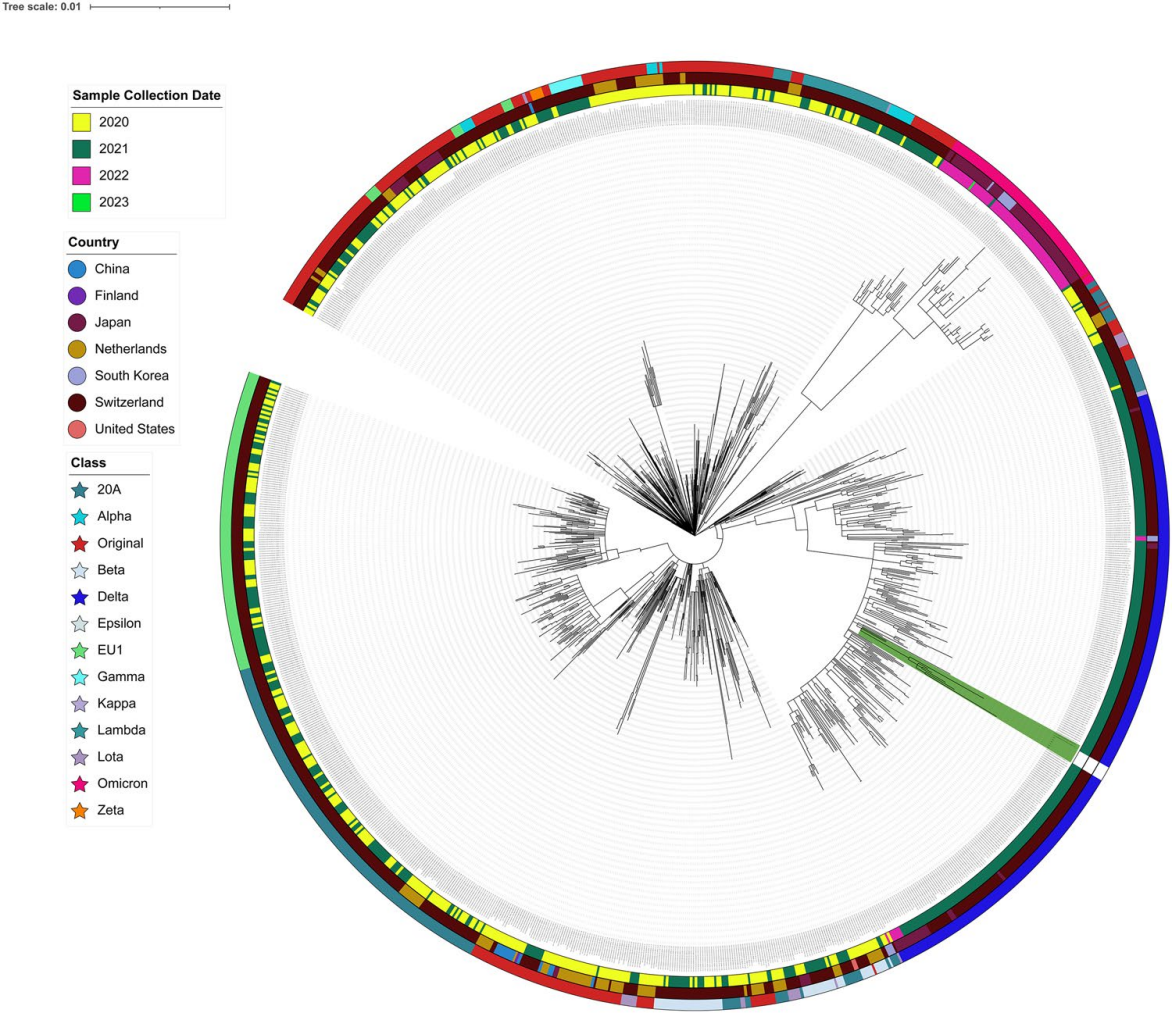
Phylogenetic tree depicting the evolutionary relationships of the six samples isolates

**Fig. 4:**
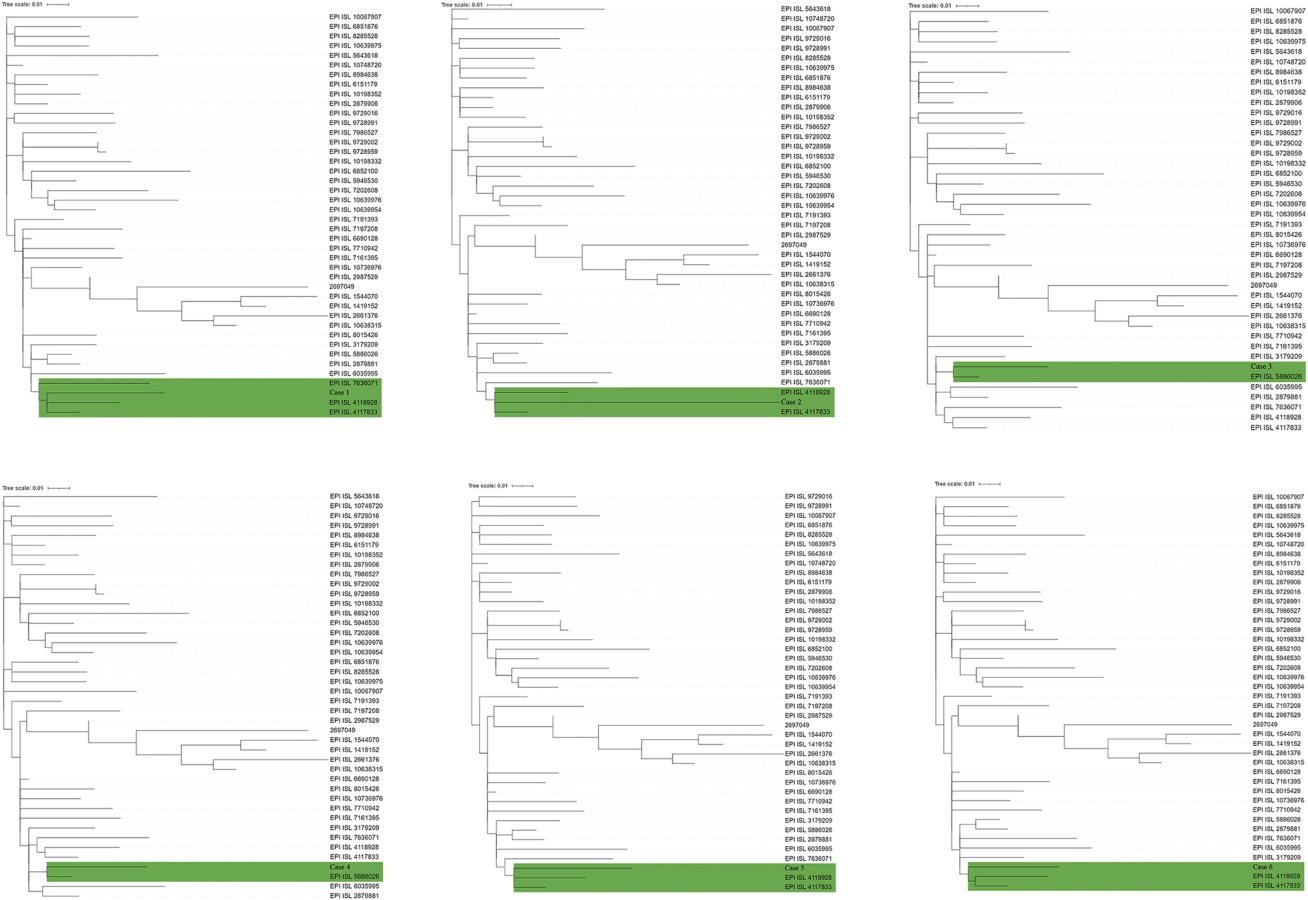
Individual phylogenetic tree analysis of 6 samples

The genomes of SARS-CoV-2 isolates from the six infected patients were investigated by next-generation sequencing. After filtering, the total reads of the isolates were aligned against the reference genome (NC_045512). Phylogenetic analysis (Fig. 3) showed that the new isolates were closely related to strains EPI_ISL_7636071 (AY.122) (Pango v.3.1.20 2022-02-02) Delta (B.1.617.2-like) (Scorpio)), all of which had been derived from Delta variant of SARS-CoV-2. In order to further verify which variant the sample belongs to, six samples were identified separately, and the results showed that they all belong to Delta variants, and were closer to AY.122 sub-lineage (Fig. 4).

## Molecular analysis of SARS-CoV-2 isolates from the six patients

**Fig. 5:**
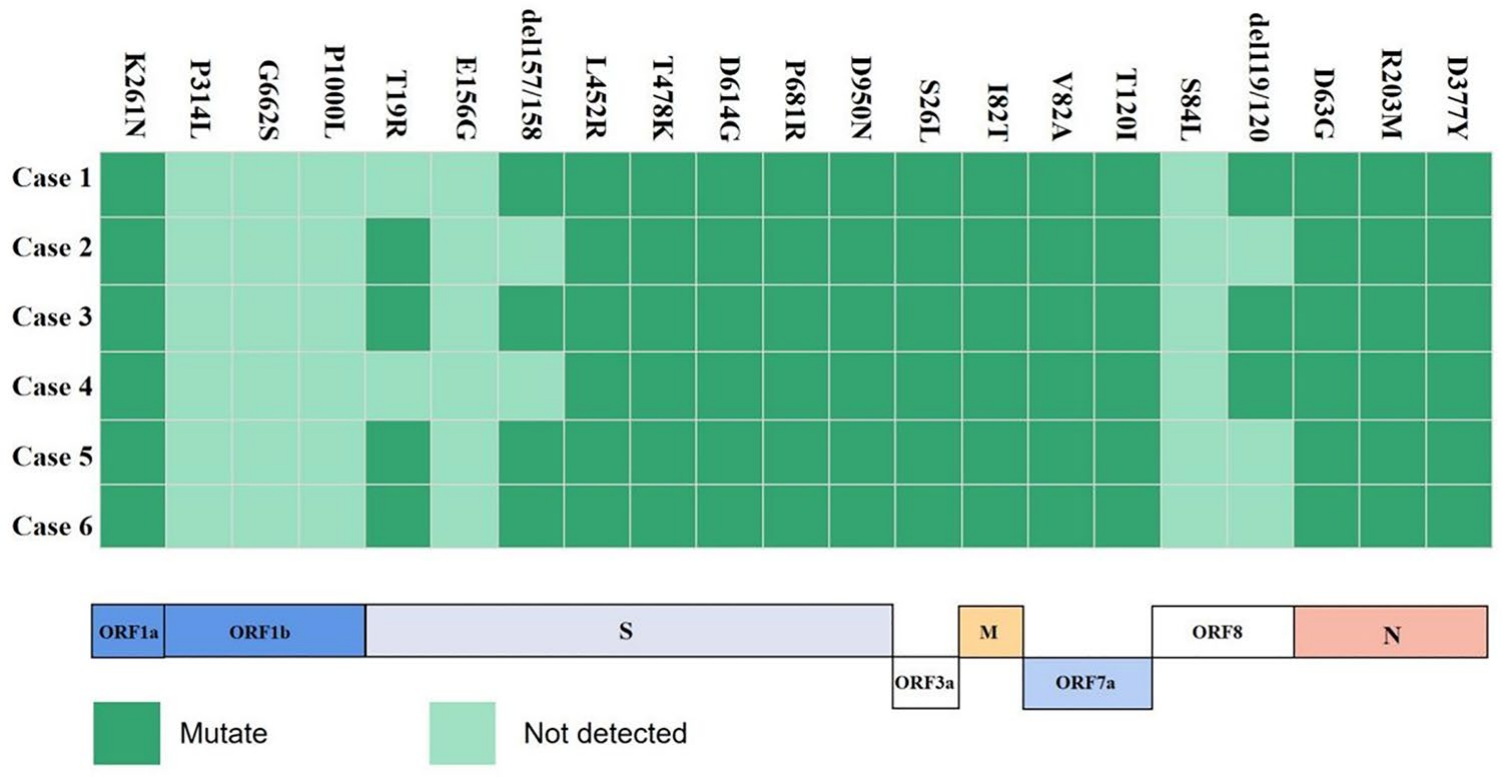
Representative mutation sites in the six new SARS-CoV-2 isolates

The result of mutation site analyses involving six SARS-CoV-2 isolates were shown (Fig. 5). The virus was identified as a subvariant of Delta (B.1.617.2) based on phylogenetic and mutation site analysis of viral sublineages revealed close similarity between AY.122 and AY.127. To characterize the sublineages of our six isolates, we searched the annotations of these two viruses on the Cov-lineages website. We found that ORF1a (K291N) is a specific mutation in AY.122, while S (T95I), ORF3a (G49V), and ORF3a (T221K) are specific mutations in AY.127. The ORF1a (K291N) mutation was present in all six isolates, thus they were assigned to the AY.122 lineage.

## Discussion

The clusters of SARS-CoV-2 infections and rapid spread of COVID-19 attracted the attention of the Inner Mongolia government, it should be immediately implemented emergency measures to reduce the risk of transmission. Among the six positive patients, three had mild disease, two had moderate disease, and one had severe disease because of underlying chronic diseases (i.e., hypertension and coronary heart disease). Although epidemiological investigations revealed that most patients had mild diseased during the current outbreak, SARS-CoV-2 infection continue to spread worldwide. These have led to additional increases in the number of confirmed cases and deaths with the emergence of new viral variants. Omicron and its subvariants have become the main pandemic strains. However, the virus identified in Inner Mongolia was a subvariant of B.1.617.2 (Delta).

The clinical findings in the six patients included various degrees of liver damage during treatment, which has attracted interest from researchers. As reported in the literature, 14–53% of COVID-19 patients have liver dysfunction, but the etiology of the dysfunction (e.g., virus or treatment) was unclear [16, 17]. This phenomenon was also reported in Cai et al. [18], in their study, 76.3% of COVID-19 patients had abnormal liver test results, while 21.5% developed liver damage during hospitalization. Among the treated patients, one developed severe liver function abnormalities after 1 week of hospitalization. We suspected that the liver injury was caused by the traditional Chinese medicine prescription (*Amomum tsao-ko*, *Areca catechu*, *Mangnolia officinalis*, *salt-treated anemarrhena rhizome*, *Scutellaria baicalensis*, *Paeoniflorin, liquorice*). The medicine was discontinued when the patient's condition improved, then the liver function gradually returned to normal. Thus, patient may have abnormal liver function during treatment, they should undergo frequent monitoring to ensure appropriate countermeasures can be performed in a timely manner. Another finding showed in our study that the six patients were cured within two weeks on average. As COVID-19 continues to develop, therapeutic schemes for various SARS-CoV-2 variants are constantly present and cure rates have increased. Aslam et al. [19] reported in current research on Delta virus, that 1640 patients were cured within 15 days, vaccinated patients have shorter hospital stay for survival outcome. A study in Singapore found that while vaccinated and unvaccinated patients infected with the Delta variant had similar viral loads at diagnosis, vaccinated viral loads declined more rapidly, and the patients have less cough/loss of smell/cold-like symptoms [20, 21].

### Analysis of major variation sites in six samples

In this study, next-generation sequencing showed that the all six isolates were related to the Delta variant, which was originally identified in India in December 2020 [22]. The statistical analysis of mutation sites revealed that the main mutations were in the spike protein, consistent with the findings of Singh [23]. Mutations in the spike protein have enhancing effects on viral load and disease spread. Adaptive genomic mutations can alter viral pathogenicity. Notably, a single amino acid exchange can greatly affect immune evasion, it can hinder the progress of vaccine development or reduce invasiveness. Similar to other RNA viruses, SARS-CoV-2 undergoes gradual genetic evolution during adaptation to new human hosts, this leads to the emergence of variants that may considerably differ from ancestral strains [24].

The Delta variant exhibits numerous mutations of S protein (e.g., T19R, E156G, del157/158, L452R, T478K, D614G, P681R, and D950N). The D614G mutation was the first mutation identified in a variant of Wuhan SARS-CoV-2 strain. This mutation is located distal to the furin cleavage site and improves the efficiency of viral cell entry efficiency by increasing affinity for the ACE2 cell-surface receptor [25], it can result in an increased SARS-CoV-2 load in the upper respiratory tract, as evidenced by the computed tomography values in our patients. A similar study showed that the 3.5-fold increase in B.1.617.2 infectivity could be attributed to the L452R mutation in the spike protein [26]. The L452R mutation enhance spike protein affinity for ACE2 and protects the Delta variant from contact with CD8+T cells, which increases viral transmissibility and infectivity [27]. These effects are consistent with our findings, as well as the COVID-19 case statistics in Inner Mongolia that indicate spread and high transmissibility. In the present study, the T478K mutation was present in all isolates, which is consistent with global mutation trends. Foreign importation and cold-chain transportation may contribute to this phenomenon [28]. Notably, the T478K mutation is the best screening target for identification of the Delta Variant [29]. The P681R mutation is another important component of the Delta variant, the amino acid substitution at residue 681 alters the cleavage site of the viral fusion protease, thereby enhancing replication [30]. This facilitates viral fusion and integration into host cells, and it might explain the high viral load in our patients.

Clinical assessment revealed that five of six patients, had completed vaccination in 2021. There is substantial evidence that antibodies produced after vaccination can neutralize the Delta variant. Data from global disease monitoring and infection surveillance programs data in 2021 showed that vaccination reduced the incidence of SARS-CoV-2 infection, as well as the incidences of hospitalization and mortality [31]. Thus, early vaccination is effective for the management of viral spread.

## Conclusion

Our study found that the virus caused this epidemic in Inner Mongolia belongs to the Delta virus AY.122 lineage. Compared with reference genome, there are some mutation sites in the S protein region, which may be the reason for the increased virulence and transmission ability of the SARS-CoV-2. We also found that some of the patients infected with Delta virus had a certain degree of liver damage, thus we should timely detect the abnormal condition of the patient's test indicators during the treatment process and correctly guide the medication. The greatest limitation of this study was its small number of patients. Although more detailed immunological indicator data were recorded, the intra-group variation was substantial; thus, we could not conduct a systematicanalysis of immunological indicators. In subsequent studies, we plan to include additional patients to minimize the potential for measurement errors. The functions of mutation sites should be investigated in greater detail.

### Sequence data

Whole-genome sequences of the six samples were submitted to NCBI database under accession number SRR21312648 to SRR21312666.

## Supplementary Information

Below is the link to the electronic supplementary material.

Supplementary file1 (XLSX 55 KB)

Supplementary Table S2 Laboratory findings for six patients with SARS-CoV-2 infection 1 (DOC 110 kb)

## Data Availability

All datasets are available in the main manuscript. Derived data supporting the findings of this study are available from the corresponding author upon request.
