# Supplementary material for: Comprehensive analysis of clinical indications and viral strain variants among patients infected with SARS-CoV-2 in Inner Mongolia, China
Source: Virus Genes. 2023 Mar 11;59(3):391–8. doi: 10.1007/s11262-023-01986-0 (PMC10006559; doi:10.1007/s11262-023-01986-0)
Supplement: Supplementary file 2 — Supplementary Table S2 Laboratory findings for six patients with SARS-CoV-2 infection 1 (DOC 110 kb) [file 11262_2023_1986_MOESM2_ESM.doc]

Supplementary Table 2. Laboratory findings for six patients with SARS-CoV-2 infection.

|  |  | Case 1 | Case 2 | Case 3 | Case 4 | Case 5 | Case 6 |
| --- | --- | --- | --- | --- | --- | --- | --- |
| SARS-CoV-2 NAT | ORF1ab | ＋ | ＋ | ＋ | ＋ | － | ＋ |
|  | N gene | ＋ | － | ＋ | ＋ | ＋ | ＋ |
|  | E gene | ＋ | ＋ | ＋ | ＋ | ＋ | ＋ |
|  | 2019-nCoV-RNA | ＋ | ＋ | / | / | ＋ | ＋ |
| SARS-CoV-2 AT (<1.0 S/COI) | SRAS-CoV-2 antibody | 0.03 | 5.09 | 12.62 | 2.06 | 1562.69 | 31.69 |
|  | IgM | 0.1 | 0.61 | 1.44 | 0.44 | 16.31 | 2.45 |
|  | IgG | 0.16 | 0.06 | 0.07 | 0.1 | 1.6 | 0.09 |
| Influenza virus IgM AT | FluA-IgM | － | － | － | － | － | － |
|  | FluB-IgM | － | ＋ | － | － | － | － |
|  | PIV-IgM | － | － | － | － | － | － |
| Respiratory pathogen IgM AT | MP-IgM | － | － | － | － | － | － |
|  | Cnp-IgM | － | － | － | － | － | － |
|  | RSV-IgM | － | － | － | － | － | － |
|  | ADV-IgM | － | － | － | － | － | － |
|  | CoxB-IgM | － | － | － | － | － | － |
| Routine blood tests | White blood cell count | 3.44 ↓ | 3.93 | 4.08 | 5.15 | 6.72 | 2.77 |
|  | Red blood cell count | 4.07 | 5 | 4.56 | 5.88 ↑ | 4.72 | 4.07 |
|  | Hemoglobin | 130 | 136 | 147 | 184 ↑ | 159 | 125 |
|  | Hematocrit | 37.8 | 39.7 | 42.5 | 52.1 ↑ | 46.2 | 37.9 |
|  | Mean corpuscular volume | 92.9 | 79.4 ↓ | 93.2 | 88.6 | 97.9 | 93.1 |
|  | Mean corpuscular hemoglobin | 31.9 | 27.2 | 32.2 | 31.3 | 33.7 | 30.7 |
|  | Mean corpuscular hemoglobin concentration | 344 | 343 | 346 | 353 | 344 | 330 |
|  | Platelets | 159 | 184 | 195 | 193 | 216 | 210 |
|  | Neutrophil % | 69.8 | 47 | 51 | 54.3 | 64.1 | 42.6 |
|  | Lymphocyte % | 23.5 | 41.5 | 39.2 | 32.2 | 16.4 ↓ | 38.6 |
|  | Monocyte % | 6.4 | 10.4 ↑ | 8.8 | 11.7 ↑ | 12.5 ↑ | 17.7 |
|  | Eosinophil % | 0 ↓ | 0.8 | 0.5 | 1 | 6.4 | 0 |
|  | Basophilic % | 0.3 | 0.3 | 0.5 | 0.8 | 0.6 | 1.1 |
|  | Neutrophil count | 2.4 | 1.85 | 2.08 | 2.8 | 4.31 | 1.18 |
|  | Lymphocytes count | 0.81 ↓ | 1.63 | 1.6 | 1.66 | 1.1 | 1.07 |
|  | Monocyte count | 0.22 | 0.41 | 0.36 | 0.6 | 0.84 ↑ | 0.49 |
|  | Eosinophil count | 0 ↓ | 0.03 | 0.02 | 0.05 | 0.43 | 0 ↓ |
|  | Basophil count | 0.01 | 0.01 | 0.02 | 0.04 | 0.04 | 0.03 |
|  | RDW-SD | 41.7 | 37 | 41.6 | 40.7 | 42.3 | 46.8 |
|  | RDW-CV | 11.8 | 12.5 | 12 | 12.4 | 11.6 | 13.4 |
|  | Platelet hematocrit | 0.15 | 0.09 ↓ | 0.22 | 0.21 | 0.24 | 0.24 |
|  | Mean platelet volume | 9.6 | 1.07 | 11.4 | 10.9 | 11.3 | 11.3 |
|  | Platelet distribution width | 10 | 11.4 | 14.2 | 12.9 | 13.7 | 13 |
|  | Platelet-large cell ratio | 20.3 | 30.2 | 35.7 | 30.9 | 35 | 35.3 |
| Coagulation function tests | Prothrombin time | 9.9 ↓ | 10.9 ↓ | 10.9 ↓ | 11.2 | 10.8 ↓ | 9.9 ↓ |
|  | Prothrombin % | 117 | 98 | 98 | 93 | 99 | 117 |
|  | International normalized Ratio | 0.87 ↓ | 0.96 | 0.96 | 0.99 | 0.95 | 0.87 ↓ |
|  | Activated partial thromboplastin time | 42.5 ↑ | 34.3 | 34.9 | 35.3 | 34.8 | 38.1 |
|  | Fibrinogen | 2.08 | 3.27 | 2.95 | 3.54 | 3.7 | 1.94 ↓ |
|  | Thrombin time | 16.1 | 15.1 | 17 | 16.7 | 14.1 ↓ | 16.1 |
|  | D-dimer | 0.55 ↑ | 0.47 | 0.2 | 0.2 | 0.53 ↑ | 0.31 |
|  | (0-0.5 mg/mL) |  |  |  |  |  |  |
| IL-6 (<7 pg/mL) |  | < 1.5 | < 1.5 | < 1.5 | 11.92 ↑ | 15.78 ↑ | < 1.5 |
| Erythrocyte sedimentation rate (0-20 mm/h) |  | 79 ↑ | 19 | 20 | 8 | 13 | 26 ↑ |
| Procalcitonin B  (0-0.5 ng/mL) |  | 0.28 | 0.13 | 0.36 | 0.42 | 0.9 ↑ | 0.31 |
